# Health Tract, No. 240

**Published:** 1866-06

**Authors:** 


HEALTH TRACT, No. 240.

### CHOLERA PREVENTED.

There is no disease known to man, which is so easily, so certainly, and so infallibly cured, as epidemic Asiatic Cholera; nor is there any other important disease which can be so certainly and so soon discovered in any particular case. The great predominant symptom is a large, painless, weakening looseness of bowels; this is not the premonitory symptom of cholera, it is cholera begun! Quietude on a bed, composure of mind, and a plain, nutritious diet, will always arrest the disease, if these measures are adopted as soon as the bowels are observed to have acted two or three times within the previous twenty-four hours.

Everything swallowed to prevent cholera, will infallibly increase the chances of an attack, because all such things are stimulant in their very nature; in this stimulated condition, the body is proof against an attack, but the moment the reaction begins to take place, the moment its effects begin to die out, that moment the system begins to go down towards the natural point, but it does not stop there, it goes just as far below that, as the stimulus raised it above, and it is at this lowered point, that the disease invades, and with a malignity, intensified in the direct proportion to the amount of stimulation.

It is everywhere known that cholera most prevails where, in common times, fevers most abound; and it is just as certainly known, that the cause of epidemic fevers is most powerful at sunrise and sunset, therefore, let persons remain in doors between those hours, which includes the time between supper and breakfast.

Cholera never attacks the body, except in its time of weakness; hence, as from the fast of the previous twelve or more hours, the body is weakened, breakfast should be taken before going outside the door in cholera times, especially as breakfast strengthens the stomach, and gives it a power of resistance against the poisonous qualities of an infected night air, and for the same reason, when the body is weak and tired by the labors of the day, it should not only be kept from the night air, but should be fortified by a warm and early supper.

Exposure to the hot sun of a summer mid-day should be avoided, nor should any labor or occupation be continued until exhaustion. The time to stop work is when the feeling of tiredness first begins to force itself upon the attention.

Eat only plain nourishing food, such as meat, bread, rice, the starches, with milk, eggs, oranges and lemons. As fruit and vegetables in cities are sure to be more or less stale before they can be used, it is better to discard them altogether.

Personal cleanliness is imperative, and scarcely needs to be insisted on But all these things are useless against uncleaned houses and yards. Each householder should make it a matter of conscience to keep his dwelling and place of business scrupulously clean from cellar to attic, and from the middle of the street to the rear line of his lot.

Do not let the mind be perplexed by questions as to the contagiousness, or portability or in infectious nature of cholera, or as to the value of a quarantine, for none of these things will, of themselves, prevent an attack of cholera in any case; but bear in mind always, that perfect and infallible exemption will be the result of personal and domiciliary cleanliness, of a plain and regular mode of living, and of a composed, cofident and fearless mind.

